# Cardiovascular Disease Mortality and Potential Risk Factor in China: A Multi-Dimensional Assessment by a Grey Relational Approach

**DOI:** 10.3389/ijph.2022.1604599

**Published:** 2022-04-29

**Authors:** Shazia Rehman, Erum Rehman, Ayesha Mumtaz, Zhang Jianglin

**Affiliations:** ^1^ Department of Dermatology, Shenzhen People’s Hospital, The Second Clinical Medical College, Jinan University, The first Affiliated Hospital, Southern University of Science and Technology, Shenzhen, China; ^2^ Candidate Branch of National Clinical Research Center for Skin Diseases, Shenzhen, China; ^3^ Department of Biomedical Sciences, Pak-Austria Fachhochschule: Institute of Applied Sciences and Technology, Haripur, Pakistan; ^4^ School of Economics, Shandong University of Finance and Economics, Jinan, China; ^5^ School of Public Administration, Hangzhou Normal University, Hangzhou, China

**Keywords:** mortality, diabetes, hypertension, CVD, high blood cholesterol, grey relational models

## Abstract

**Objectives:** This study aims to investigate the impact of hypertension, diabetes, and high blood cholesterol on increased mortality from cardiovascular diseases such as coronary heart disease, stroke, and pulmonary heart disease in a multi-dimensional way.

**Methods:** The grey relational analysis methodology is adopted to assess the connection between cardiac risk factors and related mortality. The Hurwicz and the Conservative (Min-Max) criterion approach are also utilized to identify the prospective risk factor that contributes the most to increased cardiac mortality.

**Results:** The findings reveal that hypertension has a more grounded relationship with stroke and pulmonary heart disease mortality, whereas high blood cholesterol appears to be the leading contributor to deaths from coronary heart disease. The results based on the Hurwicz and the Min-Max criterion show a robust connection between dyslipidemia, coronary heart disease, and cardiovascular disease mortality.

**Conclusion:** Combating uncontrolled blood cholesterol and blood pressure levels would necessitate a multi-pronged strategy at both the national and local levels. Besides, the suggested methodologies provide a valuable tool and additional practical knowledge for public health policymakers and decision-makers in drawing rational decisions to combat China’s rising CVD burden.

## Graphical Abstract

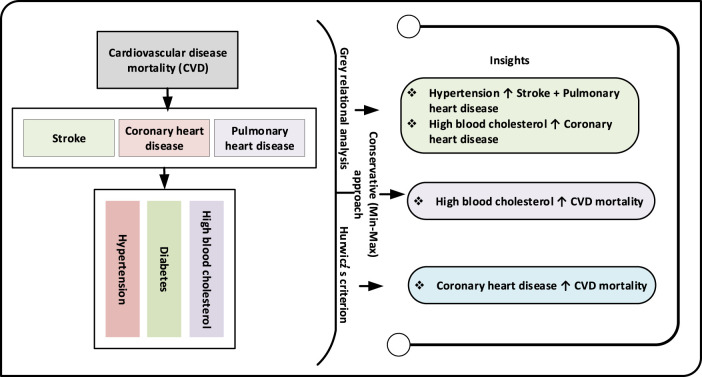

## Introduction

Cardiovascular disease (CVD) is the leading cause of death around the world, representing over 40% of deaths in China [1]. China’s tremendous economic expansion over the last few decades has brought riches and prosperity to many parts of the population. However, this progress has been accompanied by a change in disease patterns from primarily infectious to noncommunicable diseases (NCDs), with CVD being the primary cause of premature morbidity and mortality in the population of China. CVD incidence has been steadily rising, and this upward trend is expected to continue in the coming decade. The rising prevalence of CVD in China has become a serious public health concern [2, 3]. Since 2006, the prevalence of CVD in the Chinese population has been gaining momentum. Out of the 290 million patients suffering from cardiac disorders, Stroke, coronary heart disease (CHD), rheumatic heart disease (RHD), heart failure (HF), congenital heart disease, pulmonary heart disease, and hypertension (HTN) have affected 13 million, 11 million, 2.5 million, 4.5 million, 2 million, 5 million, and 245 million of individuals, respectively. In terms of mortality, CVD is responsible for two out of every five deaths in China, which is greater than the death rate from cancer or other illnesses [2, 4, 5]. Due to undertreatment and poorly managed metabolic risk factors, NCD mortality is anticipated to grow in the next decades. This may result from a worsening of metabolic risk factors, particularly, HTN, diabetes mellitus, and high cholesterol. The only approach to stop this trend is to target these risk factors through public health initiatives [6, 7].

Understanding and appropriately quantifying the impact of CVD’s key risk factors is critical for optimizing CVD prevention measures. Based on detailed literature search, for the potential risk factor analysis, we considered only HTN, diabetes mellitus, and high blood cholesterol as the top three contributors to CVD mortality in China [8–11]. Further, we included CHD, stroke, and pulmonary heart disease as the leading contributors to CVD-related mortality [10, 12–14]. The present study sought to quantify the associations of HTN, diabetes mellitus, and high blood cholesterol with mortality from stroke, CHD, and pulmonary heart disease by using grey relational analysis (GRA) models. Traditional statistical approaches, such as logistic regression, are inefficient for depicting the relations between variables in the biomedical domain, because of its dependency restrictions [15–17]. The grey relational models may overcome this shortcoming as they are devoid of assumptions. Importantly, this study employed two decision making approaches under uncertainty; 1) the Hurwicz (minimax) criteria, 2) the Conservative (minimax) criteria to conduct a comparative analysis of all three cardiac associated diseases (stroke, CHD, pulmonary heart disease) to ascertain which cardiac disorder and the risk factor is contributing more to mortality. The GRA models are being utilized for the first time in the public health domain. Hence, the current study represents first in terms of quantifying the relationship between cardiac risk factors and related mortality using grey relational techniques. Further, the suggested model provides a valuable tool and additional practical knowledge for policy and decision-makers in drawing rational decisions to reduce China’s CVD mortality rate.

## Methods

### Data Source

The data on the prevalence of HTN, diabetes mellitus, and high blood cholesterol are abstracted from Global Burden of Disease (GBD) 2017 study for the period 2001–2017. The three leading contributors of mortality from cardiac diseases in China which are stroke, CHD, and pulmonary heart disease are investigated for the study by utilizing the data from the above-mentioned website. The data are analyzed utilizing GRA methods using Grey (8.0) software. The present analysis and modeling methods are employed for the first time in the study to quantify the strength of association between cardiac risk factors and mortality from stroke, CHD, and pulmonary heart disease in a multi-dimensional way. The detailed description of the study variables is presented in supplementary file “Supplementary File S1”.

### Grey Relational Analysis Models

The GRA models, often defined as the models for grey relational analysis (GRA), are considered an early aspect of the new Grey systems theory (GST) [18]. in 1982, the concept of GST was founded on remarkable work done by a Chinese scientist, named Professor Julong Deng, and has been employed in different domains of learning since that time for dealing with vulnerability problems caused by insufficient information and mostly unclear processes. In early 1985, Professor Deng added the notion of the grey relational grade (GRG) to GRA’s model [18]. The applications of GRA models are discovered to be better and effective than other statistical models of systems analysis [19–21]. Grey relational’s core concept was to utilize an open data structure to identify whether or not their interactions are close. The algorithm involve in the calculation of grey relational analysis is presented in Supplementary File S1.

The fundamental concept of GRA is that the degree of closeness (correlation) of the geometrical structure of the data series indicates the structure variables may be used to anticipate the closeness of a relationship between the system variables. In the literature, this closeness is referred to as proximity. Deng GRA, absolute GRA, and SS GRA are the three components of the GRA model. In essence, the Deng GRA model measures the influence of one variable indicated by a data set exerts on the other, while the absolute GRA model measures the association (integral proximity) between the two. Furthermore, the second synthetic GRA model provides an estimate of an overall measure of association between the parameters under investigation. The detailed description of GRA models can be read in Liu Sifeng [22].

Traditional statistical approaches, such as logistic regression, are inefficient for depicting the relations between variables in the biomedical domain, because of its dependency restrictions. The grey relational models may overcome this shortcoming as they are devoid of dependency (normality) assumption.

Grey system theory and its model’s main strength is their ability to anticipate and make decisions with limited sample size, poor data, and missing information. The GRA models attempt to comprehend the uncertain relationships between the characteristics associated with grey systems. Unlike traditional statistical techniques, grey system theory considers all stochastic variables as a grey parameter changing within a predefined locale and a certain time frame, and each stochastic process as a grey process. Also, it provides a weighting scheme and ranking criteria, which is quite useful when several variables of equal worth are included in the analysis. This technique can also be regarded as multicriteria decision analysis and it has been applied in different domains of research so far [19, 23, 24].

Using Grey Software8.0, this study used grey relation approaches to quantify the influence of HTN, diabetes, and high blood cholesterol on CVD mortality in China from 2001 to 2017. Tables 2 –4 demonstrate the outcomes of grey relational models (Deng GRG, absolute GRG, and SSGRG) for HTN, diabetes, high blood cholesterol, and CVD mortality in China. The absolute GRG and SSGRG models have values ranging from zero to one, whereas, Deng GRG has values ranging from 0.5 to 1. It’s also considered highly associated if it’s near to 1 and weak if it diverges from 1.

## Results

In the instance of stroke, the findings revealed that all risk factors maintained their positions against all grey relational models (Table 1). The risk factor HTN (absolute GRG:.8546, Deng GRG:0.8345, SSGRG:8,446) ranked top whereas high blood cholesterol ranked second (absolute GRG:7,849, Deng GRG:.7369, SSGRG:0.7609). And the degree of association for HTN was stronger than high blood cholesterol. Considering the effect of diabetes on mortality from stroke, the estimates against absolute GRG (0.7226: rank third), Deng GRG (0.7006: rank third), and SSGRG (0.7116: rank third) models are shown to have a weak association when compared with HTN and high blood cholesterol. However, as compared to high blood cholesterol and diabetes, HTN showed a high influence on increased mortality from stroke as it stood first in all grey models. The strong connection of HTN with mortality indicated that it may be considered as the highest predictive risk factor to predict mortality from stroke in the Chinese population. Stroke is the most significant cause of death and disability in China, with around 3 million new cases diagnosed each year [10]. The strongest risk factors for overall stroke prevalence in Chinese individuals were identified to be high blood cholesterol and HTN [25, 26]. The three grey relational findings of our study are found consistent with a range of other stroke-related risk factors in China, as the burden of stroke was increasing and primary and secondary prevention is likely to be core health policy priorities in the immediate future [1, 2, 27].

**TABLE 1:** Grey relational evaluation for stroke mortality (own calculation using data from Global Burden of Disease Study, China, 2017).

Stroke	Absolute GRG	Rank	Deng GRG	Rank	SSGRG	Rank	r	Rank
HTN	0.8546	1st	0.8345	1st	0.8446	1st	0.73	1st
Diabetes	0.7226	3rd	0.7006	3rd	0.7116	3rd	0.31	3rd
High blood cholesterol	0.7849	2nd	0.7369	2nd	0.7609	2nd	0.36	2nd

Dependent variable: Stroke mortality, r: Pearson’s correlation coefficient.

In Table 2, against all three grey models, it is now obvious that high blood cholesterol appeared with the highest association and diabetes with the weakest correlation with CHD mortality. The estimation results demonstrated that the high blood cholesterol risk factor ranked top and is found to be closely related to mortality evaluated by Deng (0.8908: ranked first) and SSGRG (0.8012: ranked first) relational models. The estimated association is found to be high which suggests that high blood cholesterol risk factor has a considerable impact on CHD-related deaths in China. On the other hand, HTN (absolute GRG:0.8271; ranked first, Deng GRG:0.7011; ranked third, SSGRG:0.7641; second) and diabetes (absolute GRG:0.6625; ranked third, Deng GRG:0.7431; ranked second, SSGRG:0.7028; third) shuffled their position under all three grey relational models. In comparison to HTN and diabetes, the calculated results suggest that high blood cholesterol is an independent predictive risk factor for an increased prevalence of CHD mortality in China followed by HTN and diabetes. These findings are in contrast with some previous epidemiological studies in which HTN is identified as a significant CHD risk factor [10, 28, 29]. High blood cholesterol is a significant modifiable risk factor for CVD in China. However, the rate of treatment, control, and awareness has been estimated to be significantly below the desirable levels [30]. According to review research, if the prevalence of cardiac related risk factors continues to grow and timely attention is not made to preventive and management methods for these risk factors, CHD would soon become China’s leading cause of mortality [10].

**TABLE 2:** Grey relational evaluation for coronary heart disease mortality (own calculation using data from Global Burden of disease Study, China, 2017).

CHD	Absolute GRG	Rank	Deng GRG	Rank	SSGRG	Rank	r	Rank
HTN	0.8271	1st	0.7011	3rd	0.7641	2nd	0.41	2nd
Diabetes	0.6625	3rd	0.7431	2nd	0.7028	3rd	0.29	3rd
High blood cholesterol	0.7116	2nd	0.8908	1st	0.8012	1st	0.63	1st

Dependent variable: CHD, mortality, r: Pearson’s correlation coefficient.

Pulmonary heart disease has become the third greatest cause of mortality and disability in the Chinese population at the turn of the century [2]. Approximately 5 million of the 290 million people with CVD had pulmonary heart disease [31]. The estimated outcomes for pulmonary heart disease under the three Grey relational models are shown in Table 3. HTN (Deng GRG:0.8443, SSGRG:0.8151) was shown to be highly associated with mortality from pulmonary heart disease against absolute and Deng GRG models and ranked top as compared to diabetes and high blood cholesterol. These findings suggest that HTN has a considerable influence on the prevalence of pulmonary heart disease. Furthermore, diabetes (absolute GRG: 0.7903, Deng GRG:0.6533, SSGRG:0.7218) and high blood cholesterol (absolute:0.7853, Deng GRG:0.6529, SSGRG:0.7241) appeared with less influence on mortality from pulmonary heart disease under all three grey relational models. HTN was shown to be a considerable predictive risk factor for increased mortality from pulmonary heart disease in the Chinese population. The prevalence of HTN is very high and increasing in China while the control rate is lower than the required level. These findings are aligned with several epidemiological kinds of research in China, which found that HTN is responsible for around 40% of CVD-related events [1, 5, 32].

**TABLE 3:** Grey relational evaluation for pulmonary heart disease mortality (own calculation using data from Global Burden of disease Study, China, 2017).

Pulmonary Heart Disease	Absolute GRG	Rank	Deng GRG	Rank	SSGRG	Rank	r	Rank
HTN	0.7859	2nd	0.8443	1st	0.8151	1st	0.71	1st
Diabetes	0.7903	1st	0.6533	2nd	0.7218	3rd	0.31	2nd
High blood cholesterol	0.7853	3rd	0.6529	3rd	0.7241	2nd	0.25	3rd

Dependent variable: Pulmonary heart disease mortality, r: Pearson’s correlation coefficient.

Based on GRA outcomes, it can be seen clearly that HTN is more predictive in stroke and pulmonary heart disease-related deaths whereas high blood cholesterol is significantly high in CHD-related mortality in the Chinese population. Table 4 addresses the ranking order of risk factors (hypertension, diabetes, and high blood cholesterol) and CVDs (stroke, CHD, and pulmonary heart disease) that contribute significantly to mortality in the Chinese population using grey relational relation models. To simplify it, each grey model’s ranking order has been established against the three most prevalent CVDs with the relevant variable that has been shown. Overall, the results revealed that HTN and high blood cholesterol are the highest predictors of death from CVDs, followed by diabetes, according to the grey analysis.

**TABLE 4:** Ranking order based on Grey relational analyses (own calculation using data from Global Burden of disease Study, China, 2017).

CVDs	Grey Relational Models	Ranking Order
Stroke	Absolute GRG	HTN > High blood cholesterol > Diabetes
—	Deng GRG	HTN > High blood cholesterol > Diabetes
—	SSGRG	HTN > High blood cholesterol > Diabetes
CHD	Absolute GRG	HTN > High blood cholesterol > Diabetes
—	Deng GRG	High blood cholesterol > Diabetes > HTN
—	SSGRG	High blood cholesterol > HTN > Diabetes
Pulmonary heart disease	Absolute GRG	Diabetes > HTN > High blood cholesterol
—	Deng GRG	HTN > Diabetes > High blood cholesterol
—	SSGRG	HTN > high blood cholesterol > diabetes

We also evaluated Pearson’s correlation coefficient to compare our findings to those of the SSGRA model. As can be seen, the sequences obtained through two different approaches are very similar. The key point of differentiation, however, was their relative strengths. Although the scales of GRA models and Pearson’s correlational coefficient are not similar, their interpretations may be compared since both scales can enable the decision-maker to determine if the relationship is poor, strong, or extremely strong.

A decision analysis was used to quantify the difference between CVD mortality and the three CVD events. For this, first of all, a decision-making strategy must be constructed before it can be used. Here, m = 3, n = 3, and output = v (A_k_, C_p_), with k = 1, 2, 3 and *p* = 1, 2, 3 respectively. Let A_1_, A_2_, and A_3_ denote CV mortality associated with stroke, CHD, and pulmonary heart disease. While N_1_ represents HTN, N_2_ represents diabetes, and N_3_ denotes high blood cholesterol. Following that, the two decision-making methodologies were used, as indicated below.

### The Hurwicz (Min-Max) Criteria

The Hurwicz (Min-Max) criterion is sometimes termed as the criterion for realism. This approach is applied to make judgments (decisions) in the presence of uncertainty. It tends to be utilized in decision-making when we are confronted with several decision choices and an unpredictable natural environment. This method utilized the steps illustrated in [33]. For the present study, we need to minimize CVD-related mortality, the decision would be defined as:

$$\min A_{k}\left\{\alpha \max N_{p}v\left(A_{k},N_{p}\right)+\left(1-\alpha \right)\min N_{p}v\left(A_{k},N_{p}\right)\right\}$$

The parameter α is known as an index of optimism, and its value varies between zero and one. Here we take α as 0.8. For a minimization approach, we get the results as follows:

$$\begin{array}{l} A1:\left(0.8\times 0.8446\right)+\left(0.2\times 0.7116\right)=0.8180\\ A2:\left(0.8\times 0.8012\right)+\left(0.2\times 0.7028\right)=0.7816\\ A3:\left(0.8\times 0.8151\right)+\left(0.2\times 0.7218\right)=0.7965 \end{array}$$

The results indicated that CVD mortality is more likely to be affected by CHD (0.7816) in the Chinese population as compared to stroke and pulmonary heart disease. Overall, the estimated outcomes of both decision-making methodologies revealed that a high incidence of CHD is closely linked to an increase in overall CVD mortality. Therefore, renewed efforts for addressing the prevention, detection, treatment, and management of CHD in Chinese people are required.

### The Conservative (Min-Max) Criteria Method

This criterion was implemented in accordance with instructions used in Prasad *et. al.* [34]. Since CVD mortality is to be minimized, a min-max criterion is adopted as shown below:

$$\min A_{k}\left\{\max A_{k}v\left(A_{k},N_{p}\right)\right\}=\min A_{k}\left\{\;\begin{array}{c} 0.8446\\ 0.8012\\ 0.8151 \end{array}\right\}=0.8012$$

The findings of the SSGRG degrees-based matrix are shown in Table 5. It illustrates the SSGRG decision matrix to identify the potential risk factor of disease mortality using Conservative (Min-Max) criterion approach. The outcome (0.8012) of the conservative (Min-Max) criterion provides a distinct perspective. Surprisingly, a robust link has been observed between high blood cholesterol risk factors and CHD patient mortality in the Chinese population. The increasing prevalence of CHD appeared to have a significant impact on high blood cholesterol when compared with the other two cardiac disorders. High blood cholesterol levels appear to be an aggravating risk factor that may contribute significantly to the increased mortality of CHD patients. This finding backs up a recent review study published in 2018, which found that CHD surpassed stroke as the main cause of deaths in 2013 in six Chinese provinces, which accounts for almost 78% of the entire population [35]. In 1990, CHD was the seventh biggest cause of early mortality, but by 2010 it had risen to second place [36]. It is expected that in the coming decades CHD is anticipated to become the top cause of mortality in China.

**TABLE 5:** The criteria action matrix (own calculation using data from Global Burden of disease Study, China, 2017).

SSGRG degrees	N_1_	N_2_	N_3_
A_1_	0.8446	0.7116	0.7609
A_2_	0.7641	0.7028	0.8012
A_3_	0.8151	0.7218	0.7241

CHD incidence and death rates in the Chinese population were extremely low in the 1980s and early 1990s. A study population conducted in China had the lowest CHD prevalence and fatality rates in the MONICA research, which contained 27 nations and 38 different populations between 1984 and 1993 [37]. The rise in the prevalence of CHD in China initiated in the early 1980s and has accelerated in the following 20 years [10]. Some epidemiological research conducted in China corroborates our findings. Between 2002 and 2012, the prevalence of elevated cholesterol levels increased from 18.6% to 40.40% [16]. According to a national study published in 2018, 8.1% of Chinese individuals have abnormal blood cholesterol levels. According to a Chinese Atherosclerotic Cardiovascular disease (ASCVD) risk management framework, 1.8% of adults have a very high risk of CVD, and about 9.4% have a high risk of CVD. As a result, an estimate of 11.1% of the population may require cholesterol-lowering therapy to lessen their CVD risk. According to this comprehensive nationwide study, the rate of therapy with cholesterol-lowering medicines is only about 5.5% among the high-risk group and almost 15% among the very high-risk group [38].

## Discussion

In China, the exponential growth in cardiac mortality has sparked widespread alarm among health experts and policymakers. The role of affluence in mortality is widely studied to prevent mortality from CV disorders. This research moves a step ahead in determining the strength of association between HTN, diabetes, high blood cholesterol, and mortality caused by CVDs such as stroke, CHD, and pulmonary heart disease from 2001 to 2017 in China. We used three Grey relational models (Deng GRG, absolute GRG, and SSGRG models) in the present study, which might be a potential replacement for traditional data analysis methods. The estimated outputs revealed that the Grey relational model approach was appropriate and well-implemented.

The findings reveal that HTN has a more grounded relationship with mortality from stroke and pulmonary heart disease, whereas high blood cholesterol appears to be the leading contributor to deaths from CHD. Besides HTN and high blood cholesterol, diabetes appeared with the least influence on mortality from CVD in our study. Furthermore, to check the robustness of the results a decision-analysis approach with two different methodologies was employed using the SSGRG model outcomes, which demonstrated a robust connection between dyslipidemia, CHD, and CVD mortality. CVD mortality is shown to be considerably impacted by CHD, with dyslipidemia contributing as the most intensified risk factor for CHD-related death in the Chinese population.

Despite many positive changes in people’s lifestyle, the prevalence and the incidence of HTN elevated blood cholesterol, and diabetes, as major CV risk factors, have steadily increased in China due to population growth, aging, unfavorable lifestyles, and ambiguous etiology of these risk factors. Patients with these illnesses require medical attention, yet they are frequently misdiagnosed, neglected, or unmanaged. China is further along in the epidemiological shift, but due to undertreatment and inadequate management, the actual population levels for high blood cholesterol and HTN are much worse, but not much different for diabetes. Given the stronger association between high blood cholesterol and CV-associated diseases [5], our findings show that high blood cholesterol maybe account for the high prevalence of CHD in China and that it is also an important cardiac risk factor in parallel with HTN. Various studies consistently show a high incidence of elevated cholesterol levels in China, while the statistics vary depending on research design, demographic, and observational periods [5, 38, 39]. In China, dyslipidemia is less frequent, although it is inadequately managed. The undiagnosed and untreated dyslipidemia presents a great potential for China as the major risk factor of CHD and stroke is preventable. The levels of blood glucose (diabetes mellitus) and blood cholesterol in the Chinese population are comparable to, or somewhat better than, those in developed economies. One explanation is that, like HTN, high blood cholesterol is less frequent, but poorly managed in China [40]. As blood cholesterol levels in the Chinese population rise, encouraging the use of effective cholesterol-lowering treatments and leading a healthy lifestyle is crucial in preventing this risk factor from contributing to future increases in incidence and death from CVDs in China.

Antihypertensive and cholesterol-lowering treatments have been shown to successfully reduce the incidence of CVD in several studies. One simulation research looked at the potential benefit of blood pressure and cholesterol management treatments in Chinese people. According to the findings, if China could successfully treat HTN, 29 million strokes, 19 million AMIs, and 9 million CVD-related deaths might be averted by the year 2030. These statistics correspond to approximately 25% of projected AMIs, strokes, and CVD deaths overall [41]. Expanded treatment along with improved control of CV risk factors have helped many developed economies reduce their CVD burden. In China and the United States (US), researchers compared the treatment, awareness, prevalence, and management of significant CVD risk factors. The Chinese population has a lower prevalence of HTN and blood cholesterol levels than the US population, but significantly poorer rates of knowledge, treatment, and management of these major risk factors. CVD mortality is expected to surge in the next decades due to the deteriorating of metabolic risk factors. The best strategy to minimize the rising burden of CVD mortality is to address these risk factors through public health programs.

Improvements in healthcare quality are frequently correlated with a country’s economic prosperity. The availability of improved diagnostic or therapeutic technologies, as well as ease of access to medicines, is critical to patients’ care, particularly in the field of heart disease. The developed economies have been fortunate in minimizing the burden of CV risk factors by incorporating HTN and elevated cholesterol levels screening and management into regular primary healthcare practice, as well as offering inexpensive and effective drugs [42]. Considering China’s health infrastructure, notably in primary care, the country has the potential to replicate the success witnessed in Western countries. Since China’s 2009 health reform, free screening and regular care of HTN have been available [43, 44]. As part of extending and strengthening primary care, initiatives are required to ramp up screening and the use of antihypertensive and cholesterol-lowering drugs. Furthermore, health care providers and primary care physicians require further training on modifiable risk factors protocol, diagnosis, and treatment. Physician education programs that include a feedback mechanism can help to minimize clinical lethargy, promote guideline adherence, and improve cardiac risk factors diagnosis and treatment [45, 46]. In the last decade, increased health insurance, a national critical medication system, and other measures have been developed to reduce financial obstacles to health care and access to inexpensive pharmaceuticals [47]. Disparities in socioeconomic level and education in China’s developed economies may play a role in CV risk factors’ measurement differences [27, 48]. As a result, developing a general public education campaign in China may also assist to increase elevated cholesterol understanding and treatment. According to studies, boosting awareness and treatment through education is likely required, but not sufficient, for China to attain better control [28, 49]. To combat uncontrolled cholesterol levels and HTN in China, a major structural transition is required. The problems might be due to a lack of screening and education, which would be reinforced by procedures and rules that assure sufficient treatment and access to inexpensive drugs.

The obvious lack of knowledge, treatment, and control of HTN, diabetes, and high blood cholesterol in the Chinese population has deep and complicated causes. The accessibility and price of pharmaceuticals at various hospitals or clinics, patient adherence to treatment, and the quality of care provided by medical practitioners may all play a role in these issues. The accessibility to drugs is of vital importance. In a survey conducted in China, researchers looked at the availability, pricing, and prescriptions of antihypertensive drugs. Only 34% of these primary-care sites had all four forms of antihypertensive medication accessible, although 92% of the sites had at least one form of antihypertensive drug based on recommended guidelines. The western part of China has the least availability of drugs in village clinics and primary-care settings [49]. More research is needed to identify the key driving causes for poorer awareness, control, and treatment rates of HTN, diabetes, and increased blood cholesterol levels, taking into account a variety of elements such as patients, health providers, and health-related regulations. Optimizing the odds of effective therapy is crucial, but so is encouraging China’s populace to adopt healthy lives on a national level.

Although various lifestyle indicators have changed in a positive direction, there are still significant gaps between guideline-recommended targets and existing levels of those indicators. A vital national strategy for the prevention of CVD and other chronic disorders should be to assist in the creation and promotion of healthy lifestyles among the Chinese population. Elevated cholesterol levels affect a large number of people who are unidentified, untreated, or uncontrolled. Substantial studies are needed to determine the primary variables that contribute to the lower rates of knowledge, provided treatment, and management of this risk factor. Identifying these highlights and their ramifications will aid in a better comprehension of China’s CVD challenges and the development of effective remedies.

Besides, the SSGRA model was found to be a suitable and effective model for grey relational evaluation of CVD mortality and associated risk factors under investigation, and it might be a better alternative to traditional data analysis methods as it is based on the GST. Also, it does not necessarily require a larger sample size to make evaluations, forecasts, or decisions; it may also function effectively with small sample size [50, 51]. Though the sample size is such an important concern in healthcare and most studies employ big samples, the technique adopted in this article might help future healthcare researchers make decisions based on limited or incomplete samples. The suggested model is applicable not only in the healthcare system but also in other domains where GST is applicable.

The path ahead to diminish the CVD burden in China is wide and meandering, yet failure is not a possibility. Talking metaphorically, a sword of Damocles looms over the Chinese people, for years to come. CVD prevention and the implementation of effective strategies to attain the goal of reducing modifiable risk factors will necessitate a concerted effort from all levels of Chinese society, including government officials, academic leaders, community health professionals, families, as well as, patients. With these efforts, we anticipate a future where the CVD epidemic is tamed.

### Limitations

The information from GBD, anyway comprehensive and effectively open to practically all nations of the world, depends on routinely accessible information from those nations and regions. Variation in the robustness of data selection and processing, as well as the credibility of the cause of death, may arise. Another drawback of the investigation is the consideration of only three cardiac risk factors which has kept our analysis traditionalist. Further studies can be made by extending the variables (risk factors) for more deep information. In addition, if the variables (risk factors) are extended, subsequent research may be conducted using more grey relational methodologies to do comparison analysis.
