# Supplementary material for: Cardiovascular Disease Mortality and Potential Risk Factor in China: A Multi-Dimensional Assessment by a Grey Relational Approach
Source: Int J Public Health. 2022 Apr 29;67:1604599. doi: 10.3389/ijph.2022.1604599 (PMC9101313; doi:10.3389/ijph.2022.1604599)
Supplement: Supplementary file 1 [file Table1.DOCX]

**Table: Description of data variables (authors description of data from Global Burden of Disease Study, China, 2017)**

| **Variable** | **Unit** | **Source** |
| --- | --- | --- |
| Hypertension | Prevalence (% of total population) | GBD |
| Diabetes mellitus | Prevalence (% of total population) | GBD |
| High blood cholesterol | Prevalence (% of total population) | GBD |
| Stroke mortality | Absolute number of deaths (both sexes) | GBD |
| Coronary heart disease mortality | Absolute number of deaths (both sexes) | GBD |
| Pulmonary heart disease mortality | Absolute number of deaths (both sexes) | GBD |
